# Opposite effects of male and female helpers on social tolerance and proactive prosociality in callitrichid family groups

**DOI:** 10.1038/srep09622

**Published:** 2015-04-16

**Authors:** Judith M. Burkart

**Affiliations:** 1 Anthropological Institute and Museum, University of Zurich, Winterthurerstrasse 190, 8057 Zürich, Switzerland

## Abstract

Across a broad variety of primate species (including lemurs, New World monkeys, Old World monkeys, and apes), proactive prosociality and social tolerance are linked to allomaternal care, reaching the highest levels in the cooperatively breeding callitrichid monkeys and humans. However, considerable variation exists within callitrichids, and the aim of this study was to identify factors that explain this variation. Male and female callitrichids pursue different reproductive strategies, leading males to play a more prominent role in allomothering. We thus hypothesised that prosociality and tolerance may be affected by group composition and sex differences. We analysed social tolerance and proactive prosociality data in 49 common marmosets and found that the number of female helpers in a group was negatively correlated with group-level prosociality and tolerance. At the individual level, rearing experience or age enhanced prosociality in male, but not in female helpers. These findings are consistent with the more ambivalent role of female helpers in infant rearing. Adding data from 5 cotton-top and 5 lion tamarins strengthened this pattern. The same factor which explains variation in prosociality and tolerance across primate species, i.e. allomaternal care, is therefore also linked to variation within common marmosets, and presumably callitrichid monkeys in general.

Callitrichid monkeys show elevated levels of social tolerance and proactive prosociality, both under naturalistic^1^,^2^,^3^,^4^,^5^ and experimental conditions^6^,^7^,^8^. Most of the time, individuals in their groups show high levels of affiliative behaviour and low levels of aggression, and they frequently share food, predominantly with immatures. Food sharing in callitrichids is unusual among primates, not only because of its high prevalence, but also because it takes the form of proactive offering. Food sharing is not only reactive, where individuals passively allow others to take their food, but also proactive, where an individual offers food to others spontaneously and in the absence of begging. Proactive food sharing is suggestive of proactive prosociality, or an interest not only in one's own, but also in others' welfare. Proactive prosociality has been demonstrated experimentally, even though marked differences between studies exist, which are partially due to methodological differences that limit the comparability of the data^9^,^10^,^11^.

Recent comparative results based on a single, standardized experimental procedure that provides comparable data, the group service paradigm, suggest that the elevated levels of social tolerance and prosociality in callitrichid monkeys are linked to their cooperative breeding system, where non-mothers provide a significant amount of infant care (i.e. allomaternal care). These data from a large number of primate species including lemurs, New World monkeys, Old World monkey and apes showed that the extent of allomaternal care predicts the extent of social tolerance and proactive prosociality^7^.

However, in addition to this inter-specific pattern, considerable variation within callitrichid monkeys can also be observed, which raises the question of which factors may explain variation in social tolerance and proactive prosociality within callitrichids. If variation in social tolerance and proactive prosociality among callitrichids is driven by the same factor as interspecific variation among primates in general, i.e. allomaternal care, we should expect distinct patterns with regard to the presence of infants in a group and with regard to group composition.

First, a callitrichid group's social tolerance and prosociality may be determined by the presence of infants. Individuals from groups with infants are engaged in daily care-giving routines, and high levels of social tolerance are crucial during such periods, for instance to ascertain safe infant transfers from one carrier to the next^12^. Since all group members can act as potential carriers, high social tolerance needs to be present at the group level at that time. Infants are also the primary recipients of proactively motivated prosocial acts under naturalistic conditions. When infants are present within a group, group members thus show increased levels of expressed prosociality on a daily basis. In common marmosets, for instance, at weeks 10–16 post partum, experimentally assessed food sharing rates peak at 53% of all food items obtained by adults, and roughly half of these transfers occur proactively^13^. Nevertheless, the presence of infants within a group can only explain a small proportion of the variation in group service performance because an infant was present in only one of the test groups tested in Ref. 7. Even though this group showed particularly high levels of social tolerance and proactive prosociality, the remaining variation remains unexplained.

A second possibility is that the variation is driven by specific classes of individuals in a group. Callitrichid groups are typically composed of a breeding pair, helpers of both sexes, and immatures. Male and female helpers, usually but not always the offspring of the breeding pair^14^,^15^, are known to pursue different strategies to obtain a breeding position. As a result of these different strategies, their contribution to infant care seems to differ accordingly^2^,^3^,^16^,^17^.

Female breeding strategies are shaped by their need for allomaternal assistance to successfully raise the offspring^17^. Even though callitrichid social systems are characterized by high flexibility and both males and females emigrate from the natal group, females are often more likely to leave their natal group in the wild^17^ and more likely to be evicted^16^,^18^. Because females critically rely on helpers for raising their offspring, this can lead to fierce competition for allomaternal assistance when multiple females breed in a group. Subordinate females may try to breed in their natal group as a secondary female, but these attempts typically trigger aggressive behaviours by the dominant females, which often result in abortion or infanticide^19^,^20^,^21^. Subordinate females are thus often expelled or leave their natal group, trying to find a breeding vacancy.

In contrast to females, the typical male breeding strategy more commonly relies on cooperation, philopatry, and the possibility of inheriting a breeding position^17^,^22^. Subordinate males' breeding attempts are implemented via extra-group copulations, which do not interfere with the interests of the breeding individuals in their own group^23^. Thus, whereas female helpers who start to engage in own breeding attempts pose a threat to the breeding female in particular and group stability in general, this is not the case for male helpers. An exception can be found in case of the loss of a breeding female, which often leads to fierce competition between males too^24^.

The divergent breeding strategies of male and female callitrichids appear to have repercussions for their contribution to infant care. In both common marmosets^25^ and tamarins^26^,^27^,^28^ the number of male, but not female, helpers in a group is typically associated with infant growth and survival in the wild. However, this more relevant contribution of males is only partially reflected in the distribution of allomaternal behaviours in male vs. female helpers, and results vary considerably between studies and species^26^,^29^,^30^.

Individual contributions to infant care have been studied most extensively for infant carrying, which has high energetic and ecological costs (e.g. reduced mobility and foraging, and higher predation risks^29^). A common pattern that has been repeatedly reported is that male helpers carry more than female helpers^27^,^30^,^31^,^32^,^33^, and that older helpers carry more than younger helpers^30^,^31^,^32^,^33^,^34^,^35^,^36^. However, some studies did not find such a pattern, as for instance in a very large sample of common marmosets (13 family groups with 113 potential carrier and 25 neonates), where males and females did not differ in their contributions and no age effect could be detected^37^. One possible explanation for this discrepancy is that male and female helpers behave differently with increasing age and experience. Female helpers may start out with a very high helping motivation that decreases over time and experience, alongside with their increasing motivation to occupy a breeding position themselves. The helping motivation in male helpers, on the other side, may be more stable over time, or even increase with age due to accumulating experience.

Evidence consistent with such an opposite effect of age and experience on carrying in male vs. female helpers has been reported for tamarins and marmosets. In cotton-top tamarins^33^, adult sons carried infants more than adult daughters, but immature daughters carried more than immature sons did, and an overview over carrying contributions over 80 births showed that male helpers tend to increase their carrying contributions over time whereas it decreases or remains stable in female helpers^30^. Finally, common marmoset males seem to show a steeper increase compared to females in carrying contribution during the transition from subadults to adults^38^, and the allomaternal responsiveness of adult, non-reproductive male common marmosets was higher in individuals with rearing experience compared to individuals without rearing experience^39^.

The analysis of spontaneously occurring helping behaviours, such as infant carrying, has several limitations as proxy to identify proactive prosociality. First, helping behaviour may serve different functions, such as an increase in the infants' fitness or the necessity to gain experience. In many callitrichid species, individuals need rearing experience as a helper to be able to successfully raise their own offspring^40^,^41^,^42^. Even though these functions are not mutually exclusive, a stronger emphasis on one over the other may lead to different behavioural trajectories over time. In particular, helping motivation may decrease over time if its primary function is gaining rearing experience.

Second, group members frequently compete over access to infants^30^,^37^,^38^, and specific female helpers are sometimes prevented altogether from establishing contact with the infants or carrying them, despite desperate attempts to do so^43^. The actual contribution may thus deviate significantly from individual helping motivation.

Third, with increasing mobility, the infants themselves start playing an active role in choosing their caregivers^30^. Finally, and most importantly, individual helping motivation may be expressed in different helping behaviours (carrying, food sharing, vigilance, see also Ref. 44) which may be traded-off against each other both at the individual level and at the group level, perhaps representing some form of division of labour^45^. Consistent with this idea, contributions can vary dramatically with family composition (e.g. reviewed in Ref. 30) and the most striking finding in all studies is that whatever the individual contributions are, together they amount to the situation that the infant is cared for appropriately. These limitations in inferring underlying motivations from naturalistically occurring helping behaviours have led to the insight that approaches to experimentally assess helping motivation are a vital complement to observational studies^46^.

The aim of this study was to evaluate whether the same factor that explains variation in social tolerance and proactive prosociality in a provisioning experiment across a large number of primate species, i.e. the amount of allomaternal care, also explains variation within the cooperatively breeding common marmosets. To do so, we analysed data from 49 marmoset monkeys from 11 family groups. The majority of the data was collected in Ref. 7, additional social tolerance data was collected from five family groups (see methods for details). The group service paradigm provides a group level measure for social tolerance and proactive prosociality. In addition, individual contributions to proactive prosociality, but not social tolerance, can be calculated^7^.

Based on the naturalistic behaviour of callitrichid monkeys, I predicted that the number of male helpers would increase prosociality and social tolerance within a group, whereas the number of female helpers would have a decreasing effect. Furthermore, I predicted that individual contributions to a group's prosociality would vary according to an individual's experience and role in the group. In particular, I expected a negative effect of experience in female helpers but a positive one in male helpers. These predictions were tested in common marmosets. In order to explore whether the marmoset pattern may more generally apply to callitrichid monkeys, I also used a second, larger data set that included two additional species of callitrichid monkeys, i.e. cotton-top tamarins (S*aguinus oedipus*, n = 5), and golden-headed lion tamarins (*Leontopithecus chrysomelas*, n = 5).

## Results

### Group-level variation in proactive prosociality and social tolerance

First, I analysed which factors predicted proactive prosociality and social tolerance at the group level. This analysis was performed twice, once for common marmoset groups only, and once for the full data set that also included the cotton-top and the golden-headed lion tamarin groups.

The results presented in Table 1 show that the number of adult male helpers had no effect on proactive prosociality and social tolerance, whereas the number of adult female helpers had a negative effect, both among the common marmoset groups as well as in the full data set.

**Figure 1:**
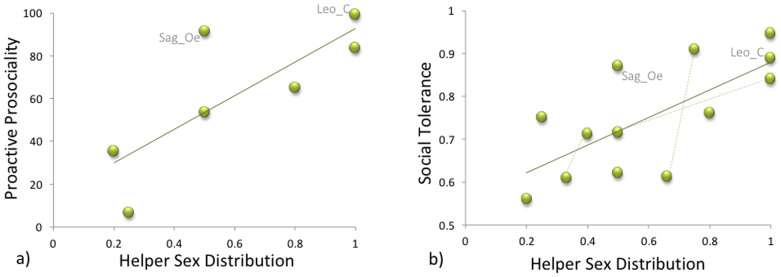
Group-level relationship between the proportion of males among helpers and (a) proactive prosociality and (b) and social tolerance. Cotton-top tamarin and golden-headed lion tamarin groups are labelled according to Table 1, all other data points represent common marmoset groups. The dotted lines in (b) connect data points from groups that have been tested a second time after the elapse of several years, after several changes in group composition had occurred.

Next, I asked whether the negative effect of female helpers was driven by the absolute number of female helpers in the group, or whether the proportion of males among helpers was more important regardless of group size. I therefore correlated both proactive prosociality and social tolerance with the percentage of males among all helpers (Table 1, last row; Figure 1a and 1b). The correlations were comparable with those of the absolute number of female helpers and based on the small sample size it was not possible to identify whether one of these variables is a better predictor.

Some groups had been tested twice for social tolerance, after the elapse of several years and after several changes in group composition had occurred. For the social tolerance analyses, I therefore included the groups that had been tested twice, as evidenced in Figure 1b. To account for the non-interdependence of these groups, I repeated the social tolerance analyses and included only the first data point for those groups that had been tested twice (i.e. Cj_Jo(a), Cj_La(a) and Cj_Ni(a)). The pattern of results remained the same. Most importantly, variation within the groups that had been tested twice was consistent with the overall trend (dotted lines in Figure 1b).

### Individual-level variation in proactive prosociality

To analyse individual variation in proactive prosociality, I used GLMMs to quantify the explanatory power of the factors *Role* (i.e. being a male or a female breeder or helper), *Experience* in rearing offspring, and *Helper sex distribution* within the group.

For the main analysis, I used a conservative data set that only contained individuals from groups who showed no ceiling effects (prosociality < 80%; individual prosociality data in the group service paradigm are most reliable in groups without ceiling effects, see also^7^). As a result of this criterion, this conservative data set only included common marmosets (*N* = 24; fb = 4, fh = 9, mb = 4, mh = 7) and we included *Group* as a random factor in the models.

To explore whether the same pattern would also hold over the entire data set, I repeated the analyses with the full data set that contained all marmosets and tamarins, and used *Group* nested in *Species* as random factor.

I calculated all main effects as well as models that contained combinations of two of the factors as well as their interaction and used the Akaike information criterion to identify the model that provided the best fit with the data. Table 2 shows that the model including the factors *Role*, *Experience*, as well as their interaction provided by far the best fit with the data, in both data sets.

To further explore the nature of this interaction, I examined each role class separately, again for the more conservative and for the full data set. These separate analyses were added to account for the fact that the number of individuals per role class was very unbalanced in the data set, because a group can maximally contain one breeder of each sex, but multiple helpers of both sexes.

**Figure 2:**
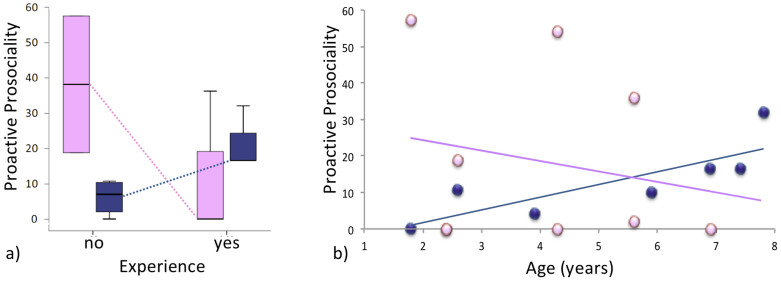
The interaction between sex and (a) rearing experience and (a) age among helpers in the more conservative data set (only common marmosets). Light pink: female; dark blue: male. The boxplots indicate medians (black horizontal lines), inter-quartile range (coloured boxes), minima and maxima (whiskers) as well as outliers (dots).

At the individual level, I found a positive effect of rearing experience on proactive prosociality in male helpers, both in the more conservative data set (GLMM, *F*_1,3.72_ = 22.5, *P* = 0.011) and in the full data set (GLMM, *F*_1,10.9_ = 6.38, *P* = 0.028, Figure 2a). In all other classes, this relationship was not significant but the correlation coefficients were negative in female helpers and female breeders (Spearman rank correlations for female breeders, conservative data set: *RS_cj_* = −0.316, full data set: *RS*_all_ = −0.339; female helpers: *RS_cj_* = −0.202, *RS*_all_ = −0.248, Figure 2a) and positive in male breeders (*RS_cj_* = 0.2, *RS*_all_ = 0.059; Table 3).

Since more experienced individuals are also older (helpers: *RS* = 0.654, p < 0.001; breeders: *RS* = 0.808, p = 0.005), I tried to disentangle whether the relevant factor that increased proactive prosociality in males was age or experience. Age was a positive predictor only in the conservative data set in male helpers (*F*_1,5_ = 9.9, *P* = 0.026, Figure 2b) and Table 3 suggests a tighter fit with experience, but the difference in AIC is small. No other significant effects were detected in any role class.

## Discussion

The aim of this study was to evaluate whether the same factor that explains variation in social tolerance and proactive prosociality across a large number of primate species, i.e. the amount of allomaternal care, also explains variation within common marmoset monkeys. Whereas common marmosets in general show high levels of allomaternal care and qualify as cooperative breeders, naturalistic observations show that there is systematic variation in individual contributions. In particular, female helpers play a more ambivalent role in infant care compared to breeders and male helpers, which is arguably reminiscent of the different breeding strategies pursued by male and female helpers.

Consistent with this more ambivalent role of female helpers in infant care under naturalistic conditions, we found opposite effects of female and male helpers on experimentally assessed social tolerance and proactive prosociality. At the group level, a higher percentage of males among the helpers of a group had a positive effect on both proactive prosociality and social tolerance. At the individual level, we found that rearing experience and age increased proactive prosociality in males but tended to decrease it in females. When the cotton-top and the golden-headed lion tamarins were added in the analyses, the results remained the same, suggesting that this pattern may not only apply to common marmosets, but to callitrichid monkeys more in general.

The interaction effect between rearing experience and sex among the helpers may help explain why studies on naturalistic helping behaviours often, but not always, find sex and experience effects: sex and experience differences may only become apparent when interaction effects are considered, in particular in large, balanced data sets (e.g. Refs. 30,37,30,37). Importantly, this interaction effect is also consistent with previous work where experimentally assessed proactive prosociality was found in male, but not in female marmoset helpers^47^. This study was composed of two family groups, and only involved adult, experienced helpers. Among experienced helpers, the interaction effect described in this study exactly predicts that males outperform females. More naturalistic data on how age and experience differentially influence the behaviour of callitrichid helpers will help to better understand this interaction effect.

Variation among callitrichids in experimentally assessed social tolerance and proactive prosociality is thus consistent with their naturalistic infant-carrying behaviour, but the current data set did not allow us to fully disentangle all potential factors, for several reasons. First, infant-carrying is only one allomaternal behaviour, but callitrichids also contribute by sharing food, vigilance and group defence^3^. We don't know to date whether all these behaviours are correlated, or whether contributions trade-off against each other within individuals and groups. We found a consistent pattern in experimental food provisioning and infant-carrying, but an integration of all helping behaviours would provide a more complete picture. Second, it is unclear whether the interaction effect between sex and age/experience is driven by age or experience, because the two are confounded in the current data set. Third, we don't know whether the sex distribution effect in group composition is driven by the negative effect of the number of female helpers in the group alone. We may not have been able to detect a positive effect of the number of male helpers because the groups did not show sufficient variation with regard to this factor (see also Table 1). Finally, it may be argued that variation in breeding strategies, which seem to be linked to variation in allomaternal care, rather than variation in allomaternal care per se explains proactive prosociality and social tolerance. Based on the available data, it is currently not possible to distinguish empirically between these possibilities. To do so, we would need individual data on allomaternal contribution as well as on the currently pursued breeding strategy (e.g. hormonal data that show whether a female helper is cycling or not).

Within the three cooperatively breeding callitrichids included in the present data set, patterns of reproductive failure suggest that *Leontopithecus* and *Saguinus* are more obligate cooperative breeders than *Callithrix* (as suggested by the necessity of helpers in the group and the importance of experience as helper to successfully reproduce^42^,^48^,^49^,^50^). In addition, *Leontopithecus* shows a more specialized form of provisioning by transporting food and putting it down directly in front of the recipient, as well as provisioning of pregnant mothers^51^ and of babysitters (unpubl. data). Consistent with these species differences, proactive prosociality was higher in *Leontopithecus* than in *Saguinus* than in *Callithrix* (Figure 1a). At the same time, the *Leontopithecus* group was the only group where an infant was present. Whereas the presence of infants had no effect on proactive prosociality across the full data set containing 15 species from all major grades of nonhuman primates (where infants were present in several groups)^7^, it is still possible that the presence of infants may have an effect within callitrichids. We are thus not able to disentangle whether the extraordinary high level of food provisioning in the golden-headed lion tamarins was exclusively driven by species differences or whether the presence of the infant had further boosted their readiness to engage in pulling food for their group members. Regardless, in either case the fundamental pattern that proactive prosociality is linked to the extent of allomaternal care holds up. However, additional data from more individuals from cotton-top and golden-headed lion tamarins, and from more callitrichid species, are needed to show to what extent this pattern applies to all callitrichid monkeys.

Female helpers are a particularly intriguing case. First of all, our results show that they don't simply lack a prosocial attitude. In fact, some female helpers showed particularly high levels of provisioning (Figure 2). This not only provides others with food, but at the same time provides them with helping experience. Furthermore, some female helpers frequently emitted submissive vocalizations while pulling the board for the others, in particular when the recipient was their mother. An intriguing possibility is thus that they did use the opportunity to instrumentally provide food to others strategically, to increase acceptance by the group and, perhaps most importantly, the breeding female. A similar argument has been made for cotton-top adult females who may have carried infants in order to avoid aggression from breeding females^52^.

Consistent with this idea, in the Cj_Jo_(a) group, the majority of all food provisioning was performed by a very young female helper, whereas her twin sister almost never did. The first individual is still part of the group today, but her twin sister was evicted and had to be removed. We therefore conducted a post-hoc analysis where we compared those helpers that stayed in the group after the experiments with those who were evicted and had to be removed (this data was available for common marmosets only, where 36.4% of the female helpers and 14.3% of the male helpers were removed). The female helpers who later had to be removed from the group virtually never provided food to their group mates and did so significantly less than other female helpers. (Mann-Whitney test, *W* = 12.5, *Z* = −2.22, *p* = 0.024). For the male helpers, no such difference was present.

Further studies will have to elucidate whether and to what extent callitrichids may be able to use prosocial behaviours strategically. Arguably, such a strategic use is most likely in female helpers, whose individual interests seem more constrained in their groups compared to the individual interests of other group members. For instance, they are often prevented by others from interacting with infants^43^ or excluded from feeding sites^53^,^54^. A particularly intriguing aspect will be whether such behaviors are regulated by cognitively demanding mechanisms or by more simple rules of thumb.

Second, the finding that the number of females decreases group-level performance in proactive prosociality and social tolerance suggests that their presence changes the dynamics in the entire group. In groups with a low proportion of males among the helpers, all group members showed less prosocial behaviour, and even the males did not take the opportunity to provide food to each other among themselves. Rather, the entire group was less likely to provide food to the others (even though the food was always taken during interspersed motivation trials where food could be made available for ego via solo effort). Groups with a high proportion of female helpers also showed lower levels of social tolerance.

One explanation for this group level effect may be that it is linked to variation in feeding competition. Variation in breeding strategies not only influences allomaternal contributions to infant care, but also feeding competition. Non-reproductive females are often excluded from feeding sites, and will seize all opportunities to get extra food^53^,^54^. A valid working hypothesis is thus that if a critical number of individuals in a group engages in fierce feeding competition, this competitive social style can spill over to the entire group.

Another explanation for the group-level effect may be that an increasing number of females among helpers presumably increases the probability of having a cycling female helper in the group. In wild lion tamarins, for instance, females that have not dispersed by 3 or 4 years of age are likely to breed in their natal group^55^. It is thus possible that the presence of a cycling helper, rather than the overall number of female helpers in the group is the relevant factor. We don't have data on the ovarian activity of most female helpers. However, we know that the group of common marmosets that had been tested twice and showed the steepest increase in social tolerance (Cj_La(a) and Cj_La(b), Figure 1b) contained a cycling helper during the first social tolerance test (Cj_La(a)). Prior to the second social tolerance test, this cycling female helper had been removed (Cj_La(b)), which led to a strong increase in the group's social tolerance. Regardless of whether the decreasing effect on social tolerance and prosociality is driven by the number of female helpers, or rather by single cycling females, it remains most remarkable that even the behaviour among the other group members is profoundly affected. Whether this situation is an artefact of captive settings remains an open question for further investigations.

In most captive settings, including our own colony, individuals are kept in their natal group as long as possible, and they are typically removed only when severe aggression occurs. Even cycling female helpers are kept in the natal group as long as the group remains stable. Even though severe aggression in captive groups is likely to reflect processes related to dispersal in the wild^56^, the present results suggest that disruptive effects on the normal functioning of callitrichid groups can emerge long before the situation culminates in escalated aggression. In the wild, in contrast, group composition is shaped by individual decisions to stay in the group or not, which may explain why many more different social systems can achieve a stable equilibrium in the wild than in captivity^57^. It is thus unclear whether captive husbandry decisions result in groups that would not persist for a long time in the wild and that are characterized by low social tolerance and prosociality. A more thorough integration of captive studies and observations from the wild may help to fully understand these processes that may ultimately lead to the observed reduction of stable social systems in callitrichids in captivity.

In sum, our findings show that as in a larger sample of primates that includes lemurs, New World monkeys, Old World monkeys, and apes, variation in social tolerance and proactive prosociality within callitrichids is linked to patterns of allomaternal care. This link is mediated by intraspecific variation in breeding strategies, which are presumably associated with patterns of allomaternal care. The results thus provide further support for the Cooperative Breeding Hypothesis for the origin of proactive prosociality^58^.

An increasing number of females among helpers, who play a more ambivalent role in infant care, decrease a groups' prosociality and social tolerance. This effect, however, can not be fully explained by lower contributions of female helpers. Rather, the higher number of females, and therefore perhaps the higher possibility of having a cycling female in the group, seems to have a disruptive effect on the prosocial efforts and dynamics of the entire group. This insight may help to refine husbandry decisions in captive callitrichids.

Finally, our results show an interaction effect between age/experience and sex on the helping motivation in callitrichids. Considering this interaction effect may help to reconcile the diverse findings with regard to sex and age differences of allocare contributions by helpers (e.g. reviewed in Ref. 30).

## Methods

Social tolerance and proactive prosociality were assessed with the group service paradigm; the data for this study were taken from Burkart et al.^7^, supplemented with additional social tolerance tests conducted with 6 common marmoset families (Table 4).

Social tolerance was assessed by sequentially offering highly preferred food items to the entire group, by placing the food item in reaching distance outside the wire mesh of the home cage of the group. For each food item, we assessed the identity of the individual who took and calculated the evenness *J*′ of this distribution^59^ as proxy for social tolerance. Low *J*′ indicates high monopolization of the food by a small number of individuals, whereas high *J*′ indicates that many group members obtain food rewards.

Proactive prosociality was assessed by offering food items on a board to the group, outside the home cage and out of reach. The board could be pulled within reaching distance by a group member, but this group member could not at the same time assess the food itself because the handle was too far away from the food and the board would pull back as soon as the handle was released. The percentage of food items made available to the group relative to all food items that were presented on the board was used as a proxy for group level prosociality.

The group service paradigm has several advantages. First, the setup is cognitively non-demanding. Second, the test does not require that individuals be separated from their group mates, which may especially affect performance in highly interdependent species. Finally, the test quantitatively assesses the degree of social tolerance and proactive prosociality as it occurs in a naturalistic situation, rather than in specific, preselected dyads.

### Subjects

13 family groups were tested, composed of 59 callitrichid monkeys from 3 species. Table 4 gives an overview of the groups and their composition. Non-weaned offspring are considered as infants, offspring older than one year as helpers. Note that three groups have been tested twice, the Jojoba-group of the common marmosets (Cj_Jo(a) and Cj_Jo(b)) with an interval of 4 years and 5 months, and the Lancia group (Cj_La(a) and Cj_La(b)) and the Nina group (Cj_Ni(a) and Cj_Ni(b)) with an interval of 2 years and 3 months. In the groups that were tested twice, the breeding individuals had remained the same but the helper composition had changed because new infants were born in the group and/or helpers had been removed. For all 13 groups, data on social tolerance was collected, whereas we also collected proactive prosociality data for 7 of these groups. Data from overlapping groups was only available for social tolerance.

The main analyses were conducted with the common marmoset data. To explore whether the marmoset pattern may also extend to other callitrichid monkeys, I used a second data set that also included the cotton-top and golden-headed tamarins. For the group-level analyses, I used the marmoset data set as well as the full data set from all callitrichids. Whereas social tolerance can only be examined at the group level, proactive prosociality can be split-up in individual contributions and used for more detailed analyses at the individual level. As a result from the group testing, this individual data is not entirely independent from each other. However, this is only the case when ceiling effects are present and food is pulled within reach in almost all trials, which potentially leaves highly motivated individuals without the possibility to provide food for others (for more details, see Ref. 7). For the individual-level analyses, I therefore used two different data sets: one that only included individuals from groups in which food provisioning occurred in less than 80% of all cases (groups with > 80% provisioning were Cj_Jo_(a), Sag_Oe, and Leo_C, see Table 3) and one that included all individuals. The first, more conservative data set only included common marmosets.

The animals were neither water nor food deprived throughout the tests. The experiments were conducted in accordance with the guidelines of the Kantonales Veterinäramt of Zurich, and all experimental protocols were approved by the Kantonales Veterinäramt of Zurich with the license numbers 4389 and 2541.

### Apparatus and procedure

The group service paradigm consists of five distinct experimental phases and has been devised to provide data on proactive prosociality and social tolerance that is directly comparable across groups and species. A detailed description of the apparatus and procedure is provided in Refs. 7,60,7,60.

**Figure 3:**
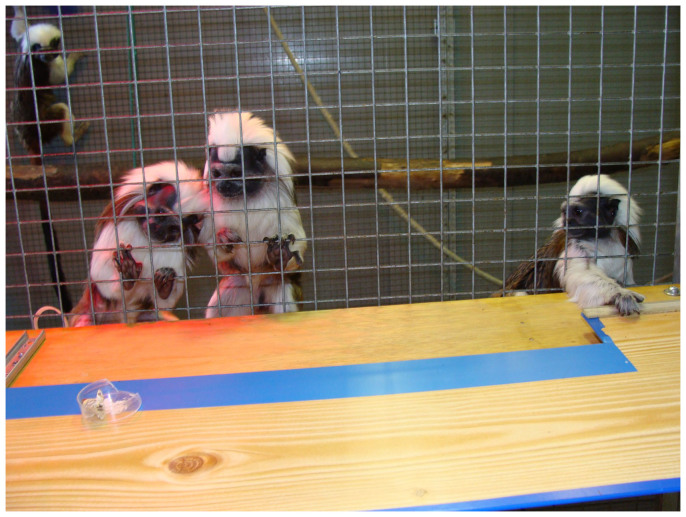
Cotton-top tamarins during the group service experiment. The individual on the right hand side pulls the board closer to the wire-mesh grid of their home cage, which brings the transparent food bowl with the food reward on top of the board within reach of its group members. The pulling individual can not at the same time take the food for itself because the food is too far away and the board draws back as soon as the handle is released. Photo by JB.

The apparatus consists of a board attached to the mesh of the home cage. A food bowl can be placed on top of the board, in various positions (Figure 3). The bowl is too far away for the subjects to reach the food. However, the board can be pulled to within reach by help of a handle on one end. As soon as the handle is released, the board rolls back because it is installed on inclined rails that run perpendicular to the mesh of the home cage. Thus, when the food bowl is placed next to the handle (during phase III of the experiment, see below), a subject can pull the handle with one hand, hold on to it, and remove the bait with the other hand. When the food bowl is placed at the other end of the board (during phase IV and V), a subject can pull the board to within reach but not at the same time reach the bait because the food bowl is too far away. In this case, the only way for anyone to acquire the food in the bowl is that one group member pulls the board, and holds it in place next to the mesh until the food is retrieved from the bowl.

The experiment consisted of five phases, and subjects had to pass predefined criteria to enter the subsequent phase. During phase I, the animals were habituated to the presence of the board and the experimenter. The board was fixed in a position next to the mesh, and pieces of favourite foods were placed in the food bowl on top of the board, within reaching distance of the subjects. Pieces of food (10 times the number of group members) were provided sequentially during 5 days, or until every subject had taken at least three pieces from the bowl. In each trial, the experimenter took a piece of food, held it up and said “look here”! in order to attract the subjects' attention. This attention-getting procedure was used in all phases of the experiment.

During phase II, social tolerance of the group was assessed. To do so, the board was still fixed in a position next to the mesh, as in phase I. On two consecutive days, 35 pieces of food were provided sequentially. We recorded the identity of the individual who took each food item and quantified the evenness of this distribution with Pielou's *J*′^59^ over both days. This index is calculated as
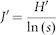
where *H'* is the Shannon Wiener diversity index:
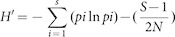
*S* is the total number of individuals in the group, and *pi* the proportion of all sequentially provided pieces of food obtained by group memper *i*. Higher values of *J*, our measure of group level social tolerance, represent a more even distribution of access to the food by the group members. The rationale for doing so was that the distribution of access to food would be more equitable among group members in tolerant groups, and more skewed toward dominant individuals in less tolerant species.

During phase III, the subjects were trained to handle the apparatus. The board was now placed at some distance away from the cage, so that the subjects could only reach the handle. The food bowl was placed one arm's length away from the handle, so that subjects could pull the handle with one hand and retrieve the food with the other hand. The training continued until all group members had done so at least seven times.

During phase IV, the board was again placed at some distance away from the cage and only the handle could be reached. In addition, the food bowl was placed more than two arms length away from the handle, so that an individual who pulled could no longer retrieve the food itself. Phase IV consisted of 5 test and 5 control sessions. Both test and control sessions were composed of 70 regular trials and 14 motivation trials (i.e. each first trial, and after every fifth regular trial). During test sessions, food was placed in the food bowl with the attention-getting procedure described above. Each trial lasted until the food was delivered to a group member or until one minute had elapsed. During control sessions, the same attention-getting procedure was used, but instead of placing a food item in the bowl, the experimenter tapped the empty bowl audibly with a stick. Only food deliveries by individuals who pulled the board significantly more often during test sessions compared to control sessions were included in the final score. During motivation trials, which were identical in test and control sessions, the bowl was placed again within one arm's length so that the food could be obtained individually. Motivation trials were included to make sure that the animals would still attend to the procedure and be motivated to get the food. Food was taken in all motivation trials in all callitrichid groups, both in control and test sessions.

During the final phase V, we added an additional control for those 5 groups who showed high levels of deliveries over all five test sessions (>40% of all trials), to exclude that transfers had occurred by mistake because the pulling subjects had not understood that they wouldn't be able to obtain the food themselves, and had not learnt this during the five test sessions in phase IV. Phase V was identical to phase IV, but the access to the food bowl in the more distant position was now prevented by a fine-meshed grid. Thus, during test sessions of phase V all visual and olfactory cues from the food in the food bowl in the more distant position were present. If pulling occurred for any reason other than providing food to group mates, e.g. due to an inability to inhibit pulling in response to salient visual and olfactory cues, the subjects should continue to pull the tray in phase V. In the Kalium group of common marmosets, instead of blocking the access with a fine-meshed grid, we moved the apparatus to the edge of the cage so that the outer part of the apparatus would extend beyond the end of the home cage. Thus, the apparatus could still be pulled but the food would nevertheless not become available to the other group members. All five groups passed phase V; Burkart et al.^7^ provides the full results from all experimental phases.

### Dependent and explanatory variables

Proactive prosociality at the group level was expressed as percentage of trials in which provisioning occurred during the last two test sessions of phase IV (see also Ref. 7). For the analysis of individual variation, I calculated individual contributions, i.e. the proportion that each group member contributed to all food deliveries of its family group. I used this relative measure of individual contributions because it controls for the substantial variation in overall deliveries between groups. Note, however, that the absolute number of deliveries per individual is highly correlated with individual contributions within the social group (r = 0.868, p < 0.001, n = 40) and both measures provide comparable results when used as dependent variable in the analyses. Social tolerance was quantified as *J*′ at the group level. Individual social tolerance levels can not be calculated in the group service paradigm.

Potential explanatory variables for group level analyses included the number of male helpers in the group, the number of female helpers, as well as the sex distribution among helpers in order to control for group size.

For the individual level analyses I used GLMMs and included age, role (i.e. female breeder, male breeder, female helper, male helper), sex distribution among helpers, and rearing experience (for helpers: the number of litters an individual had previously contributed to rear; for breeders: the number of litters reared together) as explanatory variables, as well as group or group nested within species as random factors. All statistical analyses were two-tailed and performed in SPSS 20.

## Figures and Tables

**Table 1:** Spearman correlations between proactive prosociality and social tolerance in common marmoset groups and in all callitrichid groups

		Proactive prosociality		Social tolerance	
		Callithrix	All groups	Callithrix	All groups
		N = 5 groups	N = 7 groups	N = 11 groups	N = 13 groups
# of male helpers	Rho	0.783	0.134	0.386	0.197
	p	0.118	0.775	0.24	0.518
# of female helpers	Rho	−0.872	***−0.823***	***−0.642***	***−0.696***
	p	0.054	***0.023***	***0.033***	***0.008***
Helper sex distribution	Rho	***0.900***	***0.780***	***0.758***	***0.733***
(% males among helpers)	p	***0.037***	***0.038***	***0.007***	***0.004***

**Table 2:** Fit of the GLMMs (delta AIC relative to the best model) in predicting individual prosociality in those subjects from groups without ceiling effects (CJ only) and in all subjects. In italics the model with the best fit

Model	Factors/interactions included in the model	dAIC Only callithrix	dAIC All subjects
1	[Role]	24.7	24.9
2	[Experience]	42.8	45.8
3	[Helper sex distribution]	47.3	50.9
4	[Role], [Experience]	20.4	20.5
5	[Role], [Experience], [Role]* [Experience]	***0***	***0***
6	[Role], [Helper sex distribution]	26	27
7	[Role], [Helper sex distribution], [Role]* [Helper sex distribution]	21.1	25.1
8	[Experience], [Helper sex distribution]	44.4	48.1
9	[Experience], [Helper sex dist.], [Experience]* [Helper sex dist.]	47.8	51.4

**Table 3:** Fit of the GLMMs (AIC) in predicting individual level prosociality in all subjects and in brackets in those subjects from groups without ceiling effects, which only included common marmosets (*cj*)

	Female breeders	Male breeders	Female helpers	Male helpers
	*N* = 6_all_ *(4_cj_)*	*N* = 7_all_ *(4_cj_)*	*N* = 14_all_ *(9_cj_)*	*N* = 13_all_ *(7_cj_)*
**Experience**	38.7 (n.a)	35.7 (n.a)	122.7 (72.5)	91.8*(38.0*)
**Age**	38.9 (n.a)	35.9 (n.a)	124.4 (97.2)	94.6 (42.5*)
Helper sex distribution	43.9 (n.a)	40.0 (n.a)	130.66 (103.5)	103.52 (66.16)

*: p < 0.05.

**Table 4:** Overview of the experimental groups: Type of data available and group composition

			Composition				
Species	Group	Data	fb	mb	fh	mh	infant
*Callithrix jacchus*	Cj_Jo_(a)	ST & PP	1	1	2	2	0
*Callithrix jacchus*	Cj_Jo_(b)	ST*	1	1	2	2	0
*Callithrix jacchus*	Cj_Ju	ST & PP	1	1	3	1	0
*Callithrix jacchus*	Cj_Ka	ST & PP	1	1	1	4	0
*Callithrix jacchus*	Cj_La_(a)	ST*	1	1	2	1	0
*Callithrix jacchus*	Cj_La_(b)	ST*	1	1	3	2	0
*Callithrix jacchus*	Cj_Ma	ST*	1	1	0	1	0
*Callithrix jacchus*	Cj_Mi	ST & PP	1	1	1	1	0
*Callithrix jacchus*	Cj_Ni_(a)	ST*	1	1	1	2	0
*Callithrix jacchus*	Cj_Ni_(b)	ST*	1	1	1	3	0
*Callithrix jacchus*	CJ_Vr	ST & PP	1	1	4	1	0
*Saguinus oedipus*	Sag_Oe	ST & PP	0	1	2	2	0
*Leontopithecus chrysomelas*	Leo_C	ST & PP	1	1	1	2	1

fb = female breeder, mb = male breeder, fh = female helper, mh = male helper, infant = non-weaned immature. ST = social tolerance, PP = proactive prosociality. Groups indicated by an asterix* were not part of the 2014^7^ data set.
